# Repeated exposure to aerosolized graphene oxide mediates autophagy inhibition and inflammation in a three-dimensional human airway model

**DOI:** 10.1016/j.mtbio.2020.100050

**Published:** 2020-03-24

**Authors:** L. Di Cristo, B. Grimaldi, T. Catelani, E. Vázquez, P.P. Pompa, S. Sabella

**Affiliations:** a Drug Discovery and Development Department, Istituto Italiano di Tecnologia, Via Morego 30, Genova, 16136, Italy; b Electron Microscopy Facility, Istituto Italiano di Tecnologia, Via Morego 30, Genova, 16163, Italy; c Departamento de Química Orgánica, Facultad de Ciencias y Tecnologías Químicas-IRICA, Universidad de Castilla-La Mancha, Ciudad Real, 13071, Spain; d Nanobiointeractions & Nanodiagnostics, Istituto Italiano di Tecnologia (IIT), Via Morego 30, Genova, 16163, Italy

**Keywords:** 3D airway model, Occupational exposure limits, Repeated exposure, Aerosol system, Working lifetime exposure

## Abstract

Hazard evaluation of engineered nanomaterials (ENMs) using real-world exposure scenario could provide better interpretation of toxicity end points for their use in the assessment of human safety and for their implications in many fields such as toxicology, nanomedicine, and so forth. However, most of the current studies, both *in vivo* and *in vitro*, do not reflect realistic conditions of human exposure to ENMs, due to the high doses implemented. Moreover, the use of cellular models cultured under submerged conditions limits their physiological relevance for lung exposure, where cells are primarily cultured at the air-liquid interface. Addressing such issues is even more challenging for emergent nanomaterials, such as graphene oxide (GO), for which little or no information on exposure is available. In this work, we studied the impact of repeated exposure of GO on a three-dimensional (3D) reconstruct of human bronchial tissue, using a nebulizer system focusing on short-term effects. The selected doses (reaching a maximum of ca. 20 ​μg/cm^2^ for a period of 4 weeks of exposure) were extrapolated from alveolar mass deposition values of a broader class of carbon-based nanomaterials, reflecting a full working lifetime of human exposure. Experimental results did not show strong toxic effects of GO in terms of viability and integrity of the lung tissue. However, since 2 weeks of treatment, repeated GO exposure elicited a proinflammatory response, moderate barrier impairment, and autophagosome accumulation, a process resulting from blockade of autophagy flux. Interestingly, the 3D airway model could recover such an effect by restoring autophagy flux at longer exposure (30 days). These findings indicate that prolonged exposure to GO produces a time window (during the 30 days of treatment set for this study) for which GO-mediated autophagy inhibition along with inflammation may potentially increase the susceptibility of exposed humans to pulmonary infections and/or lung diseases. This study also highlights the importance of using physiologically relevant in vitro models and doses derived from real-world exposure to obtain focused data for the assessment of human safety.

## Graphical abstract

A 3D airway model exposed repeatedly to GO for 4 weeks using doses reflecting a potential full working lifetime human exposition. Results show that exposure to inhaled GO triggered a weak proinflammatory response, impairment of barrier integrity, and autophagy blockage, thus potentially increasing susceptibility of exposed workers to pulmonary infections and/or lung diseases. Overall, the presented results highlight the importance of using physiologically relevant *in vitro* airway models and real-world exposure scenario to predict the impact of exposure to inhaled particles for human safety.

**Figure:**
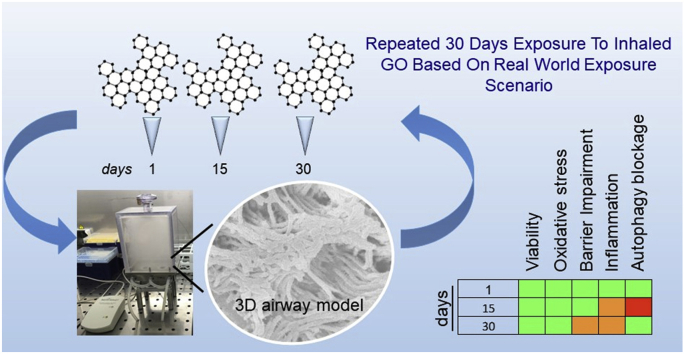

## 1 Introduction

Currently, worldwide efforts of nanosafety community are devoted to produce toxicity-oriented data to inform stakeholders (Regulatory Bodies/Policy Institutions) on the exposure limits and potential hazard effects of emergent technologies based on nanomaterials, including graphene family materials (GFMs) [1]. Graphene-based technology (energy, electronics, biomedicine, and sensors) [[2], [3], [4]] has recently raised an impressive boost [5], with a market approaching £ 300 million by 2,022 [6]. Based on these observations, the evaluation of the potential toxicity of graphene nanoforms appears urgent, as human exposure may occur (occupational, nanomedicine, consumers) [[6], [7], [8]]. For instance, the knowledge of exposure limits (e.g. occupational exposure limits [OELs]) together with other parameters of interest for nanoregulatory and/or nanomedicine (for example, Non-Observed Effect Level,NOEL, alveolar mass deposition, delivered dose, and so on) are therefore fundamental to assess occupational safety of workers, who are the first target population potentially exposed to nanomaterials, during their large-scale manufacturing. Indeed, recent studies evidenced that the air release of graphene nanoforms in production facilities can occur, raising concerns regarding the potential risks to workers' health [[8], [9], [10]]. Notably, when in powder or aerosolized forms, GO ​shows aerodynamic characteristics, which make its deposition into the deep lung regions highly possible [[3], [7], [10]]. This behavior is very similar to that of spherical nanoparticles for which this ability was widely demonstrated [[11], [12], [13]]. However, to the best of our knowledge, no official exposure limits are currently available for GFMs (including GO) [[6], [7], [8]]. As a consequence, no studies used dose values based on the potential occupational exposure of workers. In addition, considering the worker exposure (that could occur repeatedly over the entire timeframe of the working lifetime, usually estimated in the range of 30–40 years), the need for inhalation studies which could take into account a cumulative response appears clear (prolonged repeated exposure) [7]. It is worth mentioning the in vitro study of Drasler at al. [14], in which the authors have taken in consideration the exposure to GO at low, realistic doses. However, its potential toxicity effects were evaluated under an acute exposure scenario upon single aerosolization. At last, most of the in vitro data typically refer to cell responses of monoculture cellular systems at submerged conditions that do not resemble the real-world human exposure to graphene in occupational settings [15]. Overall, by analyzing the current scenario, it seems that no clear relationship between the generated toxicity data and human occupational exposure can be possible so far; therefore, no regulatory limits can be extrapolated.

In this complex framework, the assessment of repeated exposure conditions (doses repeated over long time frames, somewhat simulating the worker lifetime) by using advanced *in vitro* models, resembling tissue-like properties, would be of high relevance and could move forward the knowledge of the potential GO toxicity, providing risk assessment–oriented data. Although *in vivo* studies are potent tools for the risk assessment of nanomaterials, many ethical and economic issues must be considered [16]. Hence, the future validation of these models may provide benefits to address the reduction of animal use.

In this study, we set a 30-day exposure time, with daily treatments, to simulate a prolonged and cumulative exposure to GO over time. To reproduce more closely the physiology of a lung exposure, we used air-liquid interface (ALI) cultures, where cells are exposed to air on the apical side, whereas the basolateral side is in contact with the culture medium. As discussed previously, the selection of doses based on real-world human exposure to GFMs can represent a good approach for generation of risk assessment–oriented toxicological data [[14], [17]]. However, due to the lack of official limit values for GO, the doses selected in this work were extrapolated by read across the alveolar mass retention values that are available for the class of carbon-based nanomaterials (e.g. carbon nanotubes). These values were modeled by known OELs and were calculated to be in the range of 12.4–46.5 ​μg/cm^2^, corresponding to the possible material mass inhaled during the full working lifetime exposure of a worker (45 years) [[17], [18], [19]]. Such values are related to the mass of nanomaterial deposited on the lung surface (μg/cm^2^), so that they can be directly applied on our model, considering the mass deposition per cm^2^ of the 3D bronchial epithelium. By means of a quartz crystal microbalance (QCM), placed at the bottom of the nebulizer system (refer next paragraph for more details), the mass deposition was monitored and fixed to match the desired effective dose during the exposure. In particular, the starting GO concentration and the nebulizer setup were also settled to obtain well-defined incremental doses, which reach a maximum value that fits the aforementioned range at the end the treatment (30 days). Although the approximation about the efficiency of the mucociliary systems (that is reduced *in vitro* as compared with *in vivo*), this approach allowed the application of realistic doses of GO on an advanced in vitro airway model, mimicking a realistic scenario of human occupational exposure. Several biological end points, such as viability, inflammation, oxidative stress, membrane leakage, and autophagy, were analyzed during the entire exposure period by different analytical approaches.

## 2 Materials and methods

### 2.1 Chemicals and reagents

All chemicals and reagents used were obtained from Sigma-Aldrich (Italy), unless otherwise stated.

### 2.2 Graphene oxide

GO powder (kindly supplied by Grupo Antolin Ingeniería, Burgos, Spain) was dispersed in water and washed several times to remove the acidic residues. After lyophilization, GO powder was dispersed in a stock suspension at a concentration of 1 ​mg/mL in endotoxin-free water (#95289, Sigma-Aldrich, Italy) and sonicated by water bath for 10 ​min. The freshly prepared GO stock suspension was thoroughly characterized by high-resolution transmission electron microscopy ​JEOL 2100 ​at an accelerating voltage of 100 ​kV to provide information on morphology and lateral dimension distribution. Raman spectra were measured using an InVia Renishaw microspectrometer equipped with a 532-nm point-based laser. Thermogravimetric analysis was performed using a Themogravimetric analysis, TGA Q50 (TA Instruments) at 10 ​°C/min under nitrogen flow, from 100 to 800 ​°C [20,21]. Metallic- impurity content (μg/L) was determined using an inductively-coupled plasma mass spectrometry (ICP-MS) PerkinElmer S10 (NexION 350X) and microwave digestion (Milestone Ethos D) using concentrated nitric acid (following the ISO/DTS 13278). The digestion procedure consisted of the following microwave treatment cycle: aliquot of GO suspension (1 ​mg/mL) was warmed for 5 ​min ​at 100°C/700 ​W, for 2 ​min ​at 150°C/800W, for 5 ​min ​at 190°C/1,000W, and finally for 10 ​min ​at 190°C/700W. Inductively coupled plasma (ICP) calibration standards were used to construct a multipoint standard curve after the correction with internal standard (lutetium, 100 ​μg/L and rhodium 500 ​μg/L). The GO batch was then tested for the presence of endotoxin by the limulus amebocyte lysate (LAL) assay (Pierce, Thermo Scientific, Italy). The obtained value was 0.07±0.003 EU/mL that is below the limit of 0.5 EU/mL according to US Food and Drug Administration guidelines [22].

### 2.3 Organotypic culture of bronchial epithelial cells

EpiAirway™ tissues (AIR-100, PE6-5), a 3D mucociliary tissue model of the primary human bronchial epithelium, were purchased from MatTek Corporation (Ashland, MA, USA). EpiAirway cell cultures were positioned in a 24-well plate, where each well contains a 6.5 ​mm polyester ​Transwell® insert (surface area 0.33 ​cm^2^) on which the cells are cultured in ALI condition. Immediately after the arrival, they were placed into fresh EpiAirway™ culture medium (serum-free Dulbecco's Modified Eagle Medium, DMEM enriched with various growth factors and hormones, as provided by MatTek) (700 ​μL medium/well), inspected microscopically and then incubated with cell culture medium, reaching the 3D models from underneath at 37°C with 5% CO_2_. The day after the arrival, an apical wash with Dulbecco's phosphate-buffered saline (DPBS) was applied as suggested in the manufacturer's instructions. EpiAirway™ models were exposed to GO aerosol after two days of stabilization. Fig. S1 is reported to confirm the actual aspect of the mucociliary apparatus of the 3D model before starting the experiment. Initial transepithelial electrical resistance (TEER) values were recorded (refer following paragraph) to confirm tissue integrity. Throughout the entire course of the experiments, EpiAirway™ tissues were cultured in maintenance media (AIR-100) according to manufacturer's recommendations.

### 2.4 TEER ​measurements

Before and after repeated exposure to GO aerosol, integrity of the EpiAirway ™ model was measured by TEER using an epithelial voltohmmeter (Millicell-ERS voltmeter, Millipore). Before each measurement, the apical surface of the tissues was rinsed twice with DPBS. Fresh DPBS was added to the tissue inserts both at the apical and basolateral compartments for the measurement. The obtained resistance value was multiplied by 0.33 (surface area of AIR-100-PE6.5), resulting in final values with units of ohm (Ω)/cm^2^. The background resistance of DPBS was recorded and subtracted from all measurements. For positive control, 0.1% of Triton X-100 (24 ​h) was used ​for inducing disruption of barrier integrity. Data are expressed as percentage (%) relative to the preexposure TEER values of each tissue.

### 2.5 Human bronchial epithelial cell culture system

Human bronchial epithelial cells (Beas-2B, derived from adenovirus 12-SV40-transformed normal human bronchial epithelium), kindly provided by Dr. Bianca Maria Rotoli (University of Parma), were obtained from the American Tissue Culture Collection ​(LG Standards, England). Cells were maintained in DMEM-F12 supplemented with 10% fetal bovine serum (FBS), 15 ​mM 4-(2-hydroxyethyl)-1-piperazineethanesulfonic acid (HEPES), 100 ​μg/ml penicillin, and 100 U/ml streptomycin. BEAS-2B cells were seeded (20,000/cm^2^) into culture inserts with permeable membrane filters (pore size of 0.4 ​mm, Costar, Milan, Italy) for 24 well plates. Cells were cultured and submerged for the first week and then ​an ALI was established, removing the medium for the apical side of the insert. Cells were cultured for a further 5–7 days, and the medium (DMEM-F12 medium containing 5% FBS) was changed every other day. Cells were serum starved 24 ​h before the aerosol GO exposure.

### 2.6 Aerosol exposure system

The Vitrocell® Cloud ALI Starter Kit (Vitrocell®, Germany) was used to expose cells to GO aerosols. The system was composed of an incubation chamber in which the cells seeded in insert were exposed to GO aerosol or to negative control (endotoxin-free water). The exposure chamber contained two wells. One well is dedicated to expose cells to aerosols ​and one to assess the real-time deposition of the GO on a QCM ​(operated at 5 ​MHz, detection limit: 0.09 μg/cm2). The QCM was used to quantify the GO deposition on the insert. The aerosol is applied for a short time of 1 ​min. To generate the aerosol, the stock suspension of GO (1 ​mg/mL) was sonicated for 10 ​min in an ultrasonic bath and nebulized by means of an Aeroneb® nebulizer (span of 2.5–4.0 ​μm). This nebulizer incorporates the OnQ aerosol generator, which produces precisely controlled droplets [23]. For each aerosolization, 125 ​μL of GO suspension was added into the nebulizer unit.

### 2.7 GO deposition into the Vitrocell® Cloud

GO deposition was quantified by the incorporated QCM as described by Chortarea et al. [17] and calculated as mass per surface area (μg/cm^2^). To examine the morphology of the deposited aerosolized GO, empty transwell inserts were used and then imaged by scanning electron microscope (SEM). Samples were coated with 4-nm gold layer to improve electrical conductivity. Representative images of deposited GO and the control (no aerosolized GO) were captured using a JEOL JSM6490LA microscopy (Joel, Japan).

### 2.8 Exposure conditions and time intervals of experiments

To characterize the potential biological impact of GO in an environment that simulate the occupational setting, the 3D human airway model was exposed to aerosolized GO for up 30 days, every day. The starting GO concentration (1 ​mg/mL) and the nebulizer flux (1 ​min for nebulizing 125 ​μl of suspension) were settled to obtain different doses, ranging from the minimal of 0.71±0.05 ​μg/cm^2^ to a maximum of 21 ​μg/cm^2^. The latter concentration fits the dose range referring to the full working lifetime exposure that is 12.4–46.5 ​μg/cm^2^ [[17], [18], [19]]. Deposition of the negative control (endotoxin-free water) was below the detection limit of the QCM, which is ca. 10 ​ng/cm^2^. Cellular response after GO exposure was analyzed at several time points: after 1-3-7-10-15-20-25 and 30 days. Beas-2B cells were exposed every day to aerosol of GO for up 15 days. For autophagy flux study, bafilomycin A1 (BAF, 100 ​nM) was added in the basolateral medium 24 ​h before reaching the selected time point (15-repeated exposure times).

### 2.9 Cell morphology

Cells treated with GO or with vehicle (water) for the selected time points were fixed for 3 ​h in 2% glutaraldehyde, GTA (0.2 ​M phosphate buffer, pH 7.2), post fixed in 1% osmium tetroxide (OsO_4_) in the same buffer, and stained overnight with 1% uranyl acetate aqueous solution. Samples were then dehydrated by 5 ​min in a graded ethanol series, infiltrated with propylene oxide, and embedded in epoxy resin (Epon 812, supplied by TAABB Laboratories). For scanning electron microscopy (SEM) acquisition, samples were sputter coated on a coverslip with a 10-nm layer of 99% gold nanoparticles in an air-filled chamber and imaged using a JEOL JSM6490LA scanning electron microscope. For transmission electron microscopy (TEM) analysis, tissue sections were cut with an ultramicrotome (UC6, Leica), equipped with a diamond knife (Diatome). Images were collected with a Jeol JEM 1011 electron microscope, operating at an acceleration voltage of 100 ​kV and recorded with an 11 Mp fiber optical charge-coupled device ​camera (Gatan Orius SC-1000).

### 2.10 Resazurin assay

To assess the mitochondrial viability of cell cultures exposed to GO aerosol, the resazurin assay was used following a protocol previously published by Di Cristo et al.[23] Fluorescence measured at 572 ​nm ​was performed by means of a Tecan Spark multimode microplate reader (Tecan Italia Srl, Italy). Cell viability was calculated as a percentage (%) relative to the untreated (negative) control cell cultures. For positive controls, cell cultures were exposed to 0.1% Triton X-100 in DPBS for 24 ​h. As GO could interfere with this assay, a preliminary experiment was performed incubating the dye with diluted GO stock suspension (to reach the dose implemented for the experiments). No fluorescence signal was detected above the background signal (data not shown).

### 2.11 Lactate dehydrogenase ​release

Cell membrane damage, measurement by the release of lactate dehydrogenase (LDH) accumulated into the basolateral medium, was assessed using the CytoTox 96® Non-Radioactive Cytotoxicity Assay kit (Promega, Italy), according to the manufacturer's protocol. The Tecan Spark microplate reader was used to quantify LDH release recording the absorbance at 490 ​nm. Data are expressed as percentage (%) relative to the positive control cells. In addition, in this case 0.3% Triton X-100 in DPBS was used as positive controls. To avoid any GO interference with the colorimetric assay, each collected basolateral medium was centrifuged to pellet the possible GO passed through the transwell insert.

### 2.12 Laser scanning confocal microscopy

For laser scanning confocal microscopy (LSCM) analysis, 3D airway models were washed twice with DPBS and fixed with 4% paraformaldehyde ​for 15 ​min ​at room temperature. Cells were then permeabilized with 0.1% Triton X-100 in DPBS (1 ​h) and incubated in blocking solution (1% Bovin Serum Albumin, BSA in Dulbecco's Phosphate-Buffered Saline, DPBS) at room temperature (1 ​h). Cells were then incubated with the selected primary antibody overnight at 4°C. Anti-Mucin 5AC antibody (abcam 3649, dilution of 1:100) was used for staining globet cells, and Anti-alpha Tubulin (acetyl K40) antibody (abcam 24610, dilution of 1:200) was used for staining cilia cells. The day after, cells were washed three times with DPBS and incubated with secondary antibodies for 1 ​h ​at room temperature. Goat anti-rabbit Alexa 555 (abcam 150078, 1:400) and goat anti-mouse Alexa 488 (abcam 150113, 1:800) were the secondary antibodies applied. In the last 5 ​min of incubation, Hoechst 33342 (1:1000) was added for nuclei staining. After washing three times with DPBS, the filters were detached from the culture inserts with a scalpel blade and mounted on glass slides with transparent mounting medium (Vectashield, Vector Laboratories Inc., CA, USA) and imaged by LSCM. The analysis was carried out by a confocal microscope (Leica TCS-SP5) with an oil-immersion 63 ​× ​objective. A qualitative confocal imaging was carried out by acquiring a series of z-stack images. Surface rendering of z-stack images was carried out by ​open-source software, Nikon Software NIS-Elements. For assessing autophagy induction, cells were fixed in ice cold acetone (−20°C, for 5 ​min). Permeabilization, blocking, and primary and secondary antibody incubation were performed as described previously for mucus and cilia staining. LC3B Rabbit mAb (#3868, cell signaling, Euroclone, Italy) diluted in the ratio of 1:100 was used as a primary antibody, whereas goat anti-rabbit Alexa 555 (1:200) was used as a secondary antibody. To evaluate if the epithelial barrier was tight, cells were fixed in 100% methanol (5 ​min) at room temperature. The following steps were performed as described previously. Anti-Zonula occludens (ZO-1) protein antibody (ab216880, Abcam, UK) was used as a primary antibody (1:200), whereas goat anti-rabbit Alexa 555 (1:200) was used as a secondary antibody.

### 2.13 Cytokine ​secretion

The proinflammatory response was investigated by quantifying the accumulated amount of proinflammatory mediators, which are ​tumor necrosis factor (TNF)-α, interleukin-1β (IL-1β), interleukin-8 (IL-8), and interleukin-6 (IL-6) release into the basal medium by using the commercially available biolegend ELISA MAX™ Deluxe kits (Campoverde, Italy) according to the supplier's manual. TNF-α (10 ​ng/mL) was used as positive controls for the induction of a proinflammatory response in control tissues that were treated for 3 days basolaterally. The Tecan Spark microplate reader was used to detect the optical density at 450 ​nm. The absorbance at 570 ​nm was read and subtracted from the absorbance at 450 ​nm to obtain the corrected (blanked) values. Also in this case, to avoid any GO interference with the assay, the collected media were first centrifuged before the analysis.

### 2.14 Western blotting

Cells were lysed in Radioimmunoprecipitation assay buffer, RIPA buffer (Santa Cruz Biotechnology, Italy) and supplemented with phenylmethylsulfonyl fluoride, protease inhibitor cocktail, and sodium orthovanadate. Lysates were sonicated and centrifuged at 15,000×g for 15 ​min ​at 4 ​°C. After quantification with the Pierce® BCA Protein Assay kit (Thermo Fisher, Italy), aliquots of 20 ​μg of proteins were mixed with 4× Laemmli protein sample buffer (Thermo Fisher, Italy), warmed at 70 ​°C for 10 ​min, and loaded on a 4–12% gel for Sodium Dodecyl Sulfate Poly-Acrylamide Gel Electrophoresis (SDS-PAGE). After electrophoresis, proteins were transferred to nitrocellulose membranes (Biorad, Italy). Non-specific binding sites were blocked with incubation of 1 ​h ​at room temperature in 5% dry milk in tris-buffered saline (TBS)-Tween 20. The blots were then exposed at 4 ​°C overnight to the following antibodies diluted in 5% BSA in TBS-Tween 20: anti-GADPH (1:1000, Cell Signaling Technology), anti–heme oxygenase-1 (HO-1) (1:1000, Cell Signaling Technology), anti–NAD(P)H quinone dehydrogenase 1 (NQO1) (1:1000, Abcam, Cambridge, UK), anti–peroxiredoxin-1 (PRDX1) (1:1000, Abcam, Cambridge, UK), anti-SQSTM1/p62 (1:1000, Abcam, Cambridge, UK), and anti-LC3B (1:500, Cell Signaling Technology). After washing, the blots were exposed for 1 ​h ​at room temperature to Horseradish Peroxidase (HRP)-conjugated anti-rabbit or anti-mouse antibodies (Cell Signaling Technology), diluted 1:10,000 in blocking solution. Immunoreactivity was visualized with Lumina™ Forte Western HRP Substrate (Millipore, Italy). Relative proteins' expression levels were quantified by ImageJ software.

### 2.15 Statistical anal

Data are expressed as mean values ​± ​standard deviation and are normalized to the control untreated cells. Differences have been considered significant for p values ​< ​0.05. Statistical analysis was conducted using GraphPad Prism 6 (GraphPad Software Inc., La Jolla, CA, USA). An independent two-sided Student's t-test was performed.

## 3 Results and discussion

### 3.1 Aerosolization of GO and characterization

**Fig. 1:**
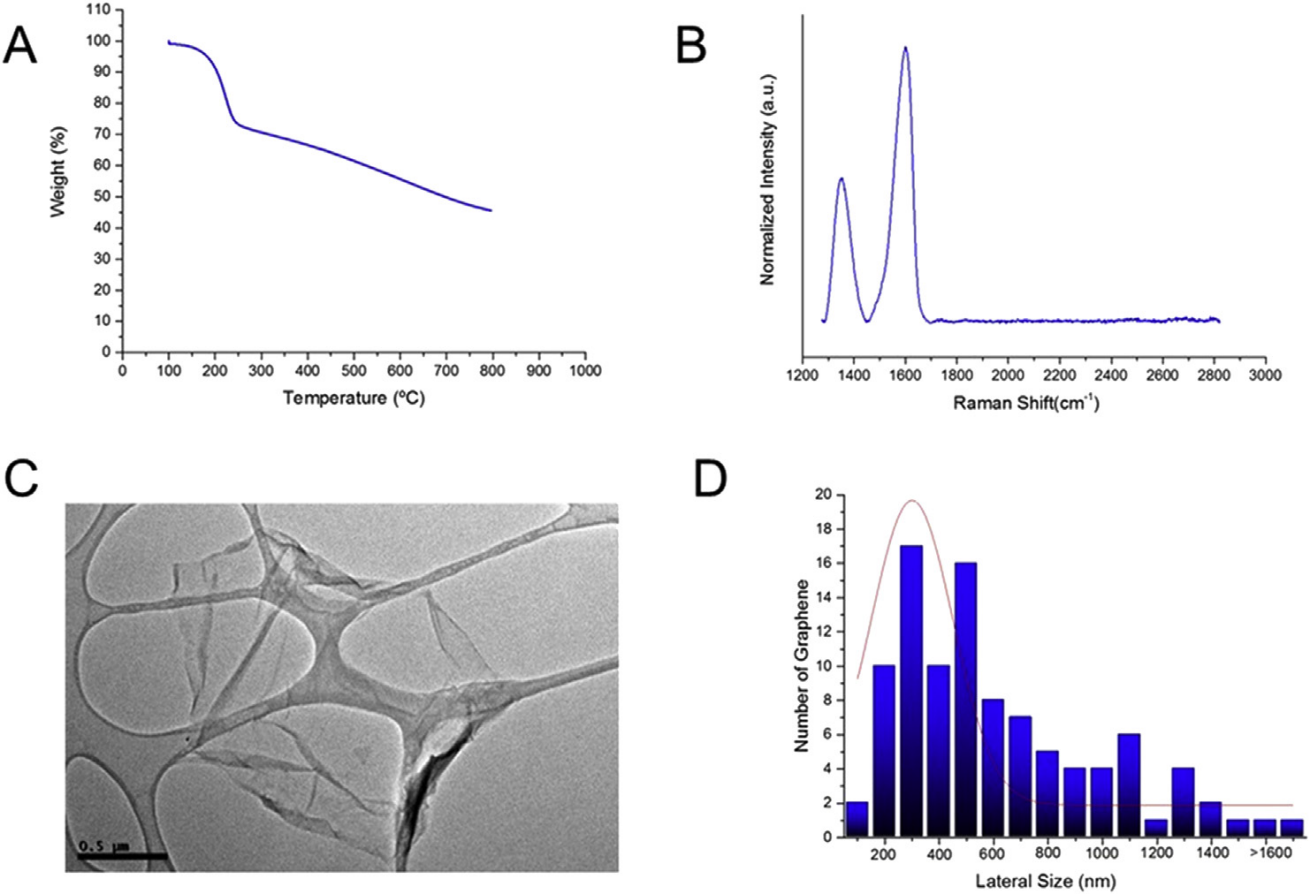
Graphene oxide characterization in water. (A) Thermogravimetric analysis of GO, (B) Representative Raman spectrum of GO, (C) Representative TEM image of an aqueous suspension of GO (scale bar 0.5 ​ ​μm), and (D) Lateral dimension distribution as measured by TEM. TEM, transmission electron microscopy; GO, graphene oxide.

A highly characterized GO material from Graphene Flagship project [20,21] was tested in this study. Physical characterization of GO in water is reported in Fig. 1. Thermogravimetric analysis showed a weight loss of 46% at 600ºC, which corresponds to the oxygen groups on the GO surface (Fig. 1A). Moreover, Fig. 1B shows the typical Raman spectrum of GO with the G peak (≈1,580 ​cm^−1^) and a rather high D peak (≈1,350 ​cm^−1^), corresponding to the defects on GO flakes. Lateral sizes of the material were analyzed by TEM, revealing a broad size distribution (100–1,500 ​nm) (Fig. 1C and D).

**Fig. 2:**
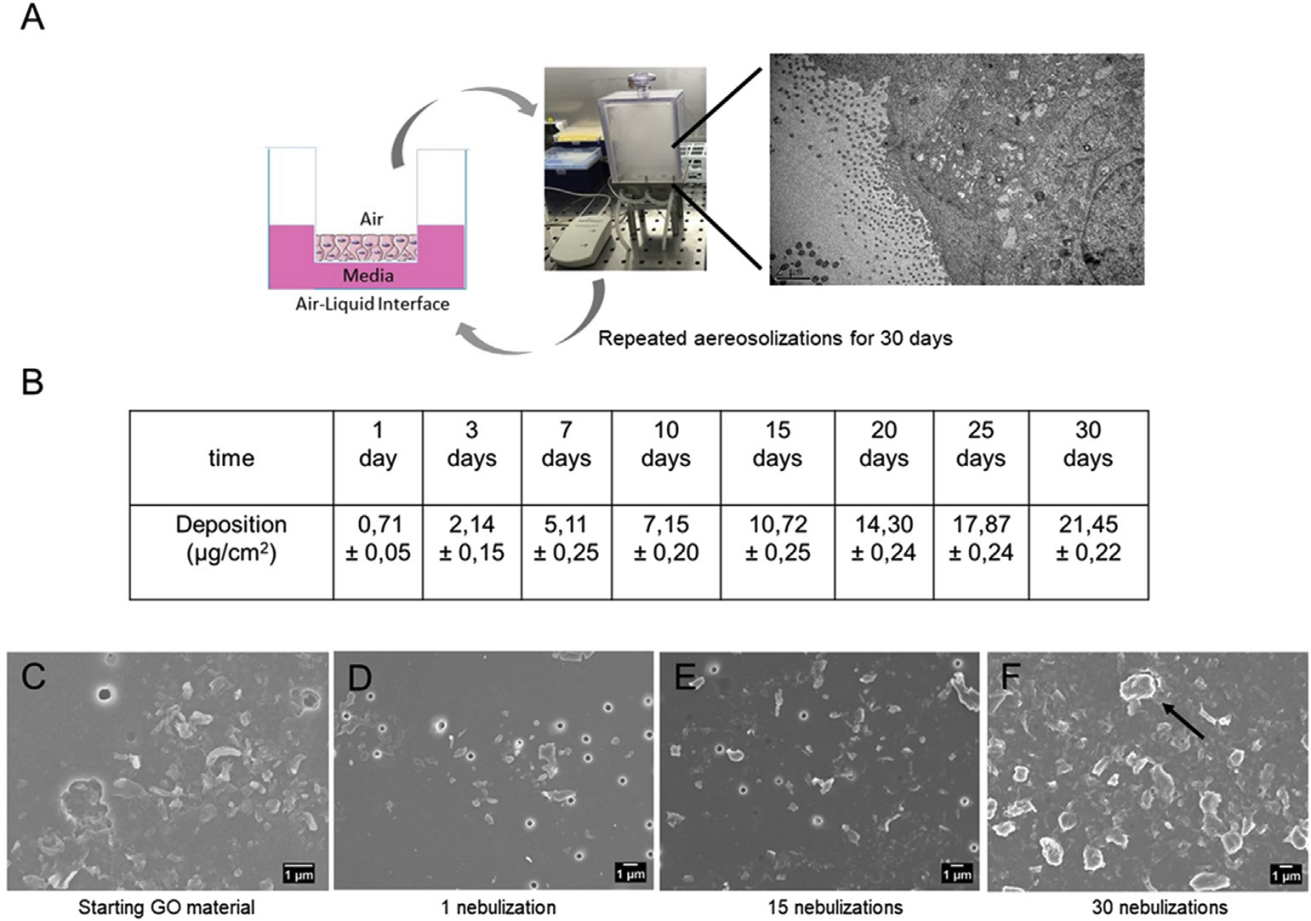
In vitro 3D airway model coupled to a nebulizer implemented in the study. (A) A three-dimensional (3D) in vitro reconstruct of primary human bronchial epithelium (also referred to as 3D airway model throughout the manuscript) cultured at ALI condition was exposed to repeated doses of aerosolized GO for up 30 days by means of Vitrocell Cloud® system. (B) Average deposition of aerosolized GO expressed in μg/cm^2^, as measured by QCM at different exposure time points (C-D-E-F). Representative SEM images of GO deposition occurred on PET transwell of starting GO material (C) and nebulized at 1- (B), 15- (C), and (D) 30-repeated exposure times, respectively. Scale bar: 1 ​ ​μm. Black arrow in panel D shows GO flakes, whereas, pores of the PET membrane are visible in the images as round structures. QCM, quartz crystal microbalance; SEM, scanning electron microscopy; GO, graphene oxide; PET, polyester.

We assessed the biological impact of low, repeated doses of GO using a nebulizer system (Vitrocell® Cloud ALI started Kit) and a D in vitro reconstruct of primary human bronchial epithelium cultured at the ALI (Fig. 2A). Vitrocell® is a well-known aerosol system used in several toxicological studies in which an ALI is needed [[14], [17], [23], [24]]. This system allows for a uniform deposition of aerosols with a precise control of the effective doses that are deposited onto the cell layer. Vitrocell® benefits of a Q CM at the bottom of the plate, allowing for an accurate monitoring of the mass deposition of the aerosolized substances. In our study, the EpiAirway™ model (refer in the following paragraphs for more details) was positioned within the Vitrocell® system, as imaged in Fig. 2A. The 3D airway model was applied for repeated exposure to GO every day, for 30 days, using doses relevant to human occupational exposure to nanocarbon materials, as no information on exposure limits for GO is specifically available [[7], [8], [9]]. The nanocarbon doses refer to the alveolar mass deposited onto the lung and were calculated to be in the range of 12.4–46.5 ​μg/cm^2^ for a full working lifetime exposure (45 years) [[17], [18], [19]]. In our experimental conditions, the starting GO concentration (1 ​mg/mL) and the nebulizer flux (1 ​min for nebulizing 125 ​μl of suspension) were optimized to obtain different doses, as reported in the table in Fig. 2B. The single GO aerosolization resulted in a deposited dose of 0.71±0.05 ​μg/cm^2^, whereas repeated GO nebulization gave deposited doses of ca. 21 ​μg/cm^2^ after 30 days (which fits the dose range described previously, referring to the full working lifetime exposure). Deposition of the negative control (endotoxin-free water) was below the detection limit of the QCM, which is ca. 10 ​ng/cm^2^. SEM ​analysis was performed on the starting GO material and on the deposited aerosolized GO structures (Fig. 2C vs. Fig. 2D–F). SEM imaging showed that the aerosolization process did not affect the morphology of the deposited materials, as no significant changes in the GO flakes were observed, compared with the starting material deposited on the filter without nebulization. Moreover, SEM and QCM analyses highlighted a dose-dependent and homogeneous deposition of the material after repeated nebulizations (Fig. 2).

The possible presence of metallic impurities in the GO suspensions (contaminants that could influence the biological outcomes) ​was evaluated by ICP-MS. The trace metal amounts, commonly present in carbon-based suspensions [25,26], are reported in Table S2. Most of these elements were found below the detection limit of the instrument. Cobalt, chromium, copper, molybdenum, lead, and tungsten ​were detected, although at low concentrations that are not relevant to cause any form of toxicity in our experimental conditions [27,28].

### 3.2 Uptake of GO in a 3D airway model upon 30-repeated exposure

**Fig. 3:**
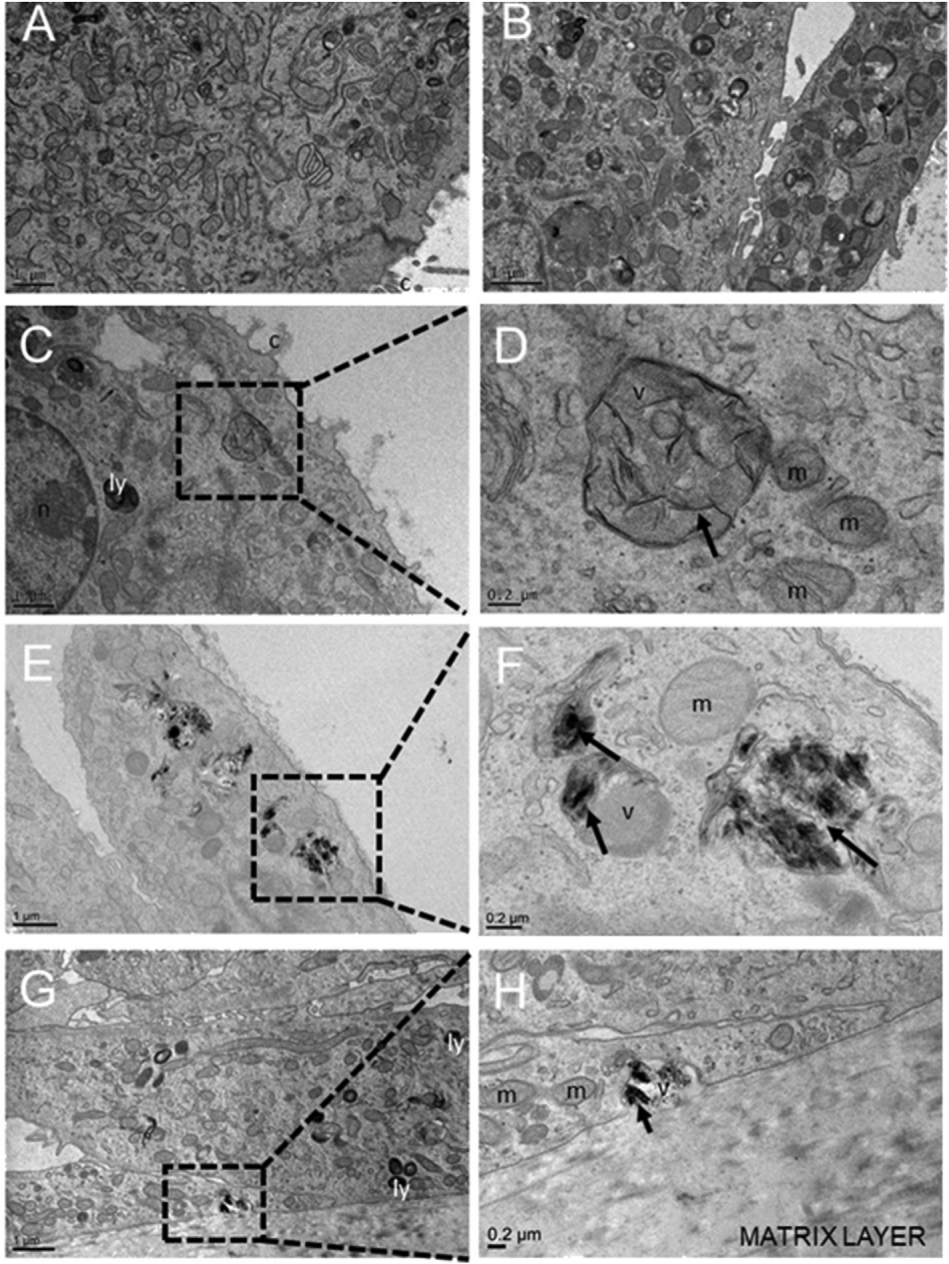
GO uptake by EpiAirway ^TM^. Representative TEM micrographs of (A) not treated tissue and tissue repeatedly exposed to GO for (B) 7 days, (C–D) 15 days, (E–F) 20 days and (G–H) 30 days. Scale bars: 1 ​ ​μm ​at lower magnification and 0.2 ​μm ​at higher magnification. Black arrows in panels D-F-H show GO single and aggregate flakes. TEM, transmission electron microscopy; GO, graphene oxide; c, cilia; ly, lysosome; v, vesicle; m, mitochondria; n, nucleus.

After inhalation, particles and microbes can deposit on the lung surface, where they interact with bronchial epithelial cells that act as first defence against xenobiotics [29]. The airway epithelium is a structural barrier that regulates water and ion transport ​and contributes to the clearance of inhaled substances through mucociliary clearance [30]. This occurs through the combined function of ciliated epithelial and secretory cells enabling efficient mucociliary clearance through a variety of host defence mechanisms [[31], [32], [33]], including the recruitment of inflammatory cells. These inflammatory cells upregulate the adhesion molecules in response to inflammatory stimuli, allowing for the adhesion of neutrophils and mononuclear cells to the inflamed area [34]. The study of defence system when exposed to subchronic or chronic insult is clearly very informative when using an *in vivo* approach. However, inhalation studies using animals are cost and time consuming, and ethical considerations associated with animal sacrifice must be carefully taken into account according to the ‘3R concept’ [35]. Therefore, the development of advanced in vitro models capable of predicting *in vivo* lung toxicity is now taking hold. Certainly, the use of 3D culture including primary macrophages would increase the quality of the model, but at this moment, the use of isolated macrophages remains critical due to their short lifetime [17]. Despite this limitation, a 3D airway model alone faithfully reproduced the native tissue representing a valid alternative to the use of animals ​because it contains important features that are valuable for inhalation safety assessment studies [[17], [36]]. In our study, we used a 3D reconstruct of the human bronchial tissue (EpiAirway™) cultured at ALI condition (also referred to as 3D airway model throughout the manuscript). This model incorporates basal cells, mucus-producing goblet cells, functional tight junctions (TJs), and beating cilia, which resemble the human epithelial barrier. The model recapitulates the *in vivo* mucociliary response to an infection or toxicants [37] and is suitable (i) to study particle/cell interactions upon inhalation at ALI condition, an environment that closely resembles the in vivo conditions and (ii) for long-term studies to simulate occupational exposure to nanoparticles. A structural analysis of EpiAirway™ exposed to repeated GO aerosolizations (0–30 days) was performed by TEM ​(Fig. 3). TEM images showed that the repeated treatments of GO did not compromise the tissue in terms of morphology and cell viability, as no significant differences were observed between the exposed tissue and the control (Fig. 3A vs. Fig. 3B–H). Images showed some intracellular GO in the form of single flakes, taken up by cells only after 15 days since the initial treatment (Fig. 3C and D). GO was distributed in large endosomal vesicles, in line with literature data reporting that GO internalization occurs primarily via endocytosis [38,39], partly operated also by non-phagocytic cells [40,41]. The number of GO-loaded vesicles increased at 20 days of treatment, showing a consistent GO structural organization as agglomerates (Fig. 3E and F). Interestingly, at the end of the exposure period (30 days), we noticed a general decrease of the GO-loaded vesicles per cell, possibly indicating an increased clearance mechanism by which the GO is trapped within the mucus and cleared away from the airways. However, the few observed vesicles were mainly located at the tissue basolateral side (Fig. 3G and H). This suggests possible vesicle translocation, resulting in some GO accumulation and therefore lack of material degradation. The absence of degradation was also recently reported by Guarnieri et al.[20], using an intestinal epithelium model. Moreover, long persistence of graphene nanoplatelets in the lung was also found *in vivo*, causing adverse health effects by disturbing immunological and physiological homeostasis [42]. Thus, this parameter ​is relevant for the toxicity evaluation of GO in real-world human exposure scenario, where indeed prolonged exposure of workers in occupational settings is likely (about 45 years). We can indeed hypothesize that the lack of GO elimination from lung regions, over the occupational worker lifetime might allow for the material translocation through the lower region of the lung, that is, the alveoli, where no protective mucus layers exist. Here, GO may be engulfed by alveolar macrophages and cleared very slowly over months or years [43]. Possible toxic effects can then be expected. In a recent review on the safety assessment of GFMs, the authors report some indication of cytotoxicity of GFMs which are related to the specific physicochemical properties of the tested materials, but further studies focused on systematic investigations looking at long-term impact of graphene are required [44].

### 3.3 Viability and integrity of a 3D airway model after 30- repeated exposure to GO

**Fig. 4:**
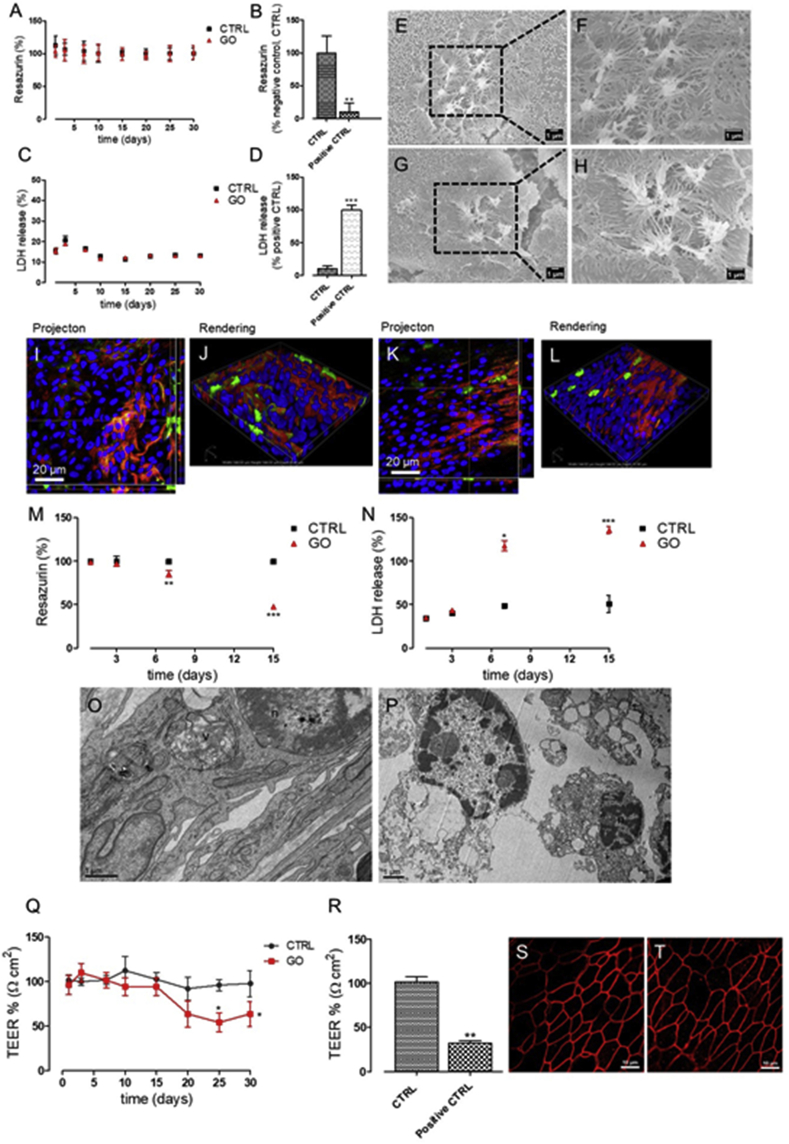
Viability and integrity of 3D airway model and BEAS-2B cells after repeated exposure to GO. (A-B-C-D) Tissue viability was assessed after 1, 3, 7, 10, 15, 20, 25, and 30 days of repeated exposure to GO aerosol using (A–B) Resazurin assay and (C–D) LDH assay. (B,D) Tissue viability treated with 0.3% Triton X-100 for 24h, used as the positive control for both the assays. Data are expressed as mean ​± ​standard deviation (n tests ​= ​3). (B) ∗∗p ​< ​0.01 vs. untreated control cells (CTRL). (D) ∗∗∗p ​< ​0.001 vs. positive control cells. (E–F) SEM micrographs of cell surface before GO exposure (G–H) and after 30 repeated exposure to GO. Scale bars: 1 ​μm ​at lower magnification and 0.5 ​μm ​at higher magnification. (I–J) Projection and rendered reconstruction of representative confocal microscopy images of human bronchial epithelial cells before GO exposure and (K–L) after 30 days of GO repeated aerosolization. Cells were stained with Hoechst 33342 (nuclei, in blue), Mucin 5AC antibody (globet cells, in red), and alpha Tubulin (acetyl K40) antibody (cilia cells, in green). Scale bars: 20 ​μm (63 ​× ​objective lens). (M) Viability of BEAS-2B cells exposed to repeated GO aerosolization for 15 days assessed with resazurin assay and (N) with LDH assay. Data are expressed as mean ​± ​standard deviation (n tests ​= ​3). (A)∗∗p ​< ​0.01 and ∗∗∗p ​< ​0.001 vs. untreated, control cells (CTRL). (B)∗p ​< ​0.05 and ∗∗∗p ​< ​0.001 vs. positive control cells. 0.3% Triton X-100 was used as the positive control (24h). TEM micrographs of (O) not treated BEAS-2B control cells and (P) GO treated BEAS-2B cells for 15. Scale bars: 1 ​μm. (Q) TEER measurements of 3D airway untreated or treated to repeated GO aerolization (1, 3, 7, 10, 15, 20, 25, and 30 days). (R) TEER measurement of 3D airway exposed to 0.3% of Triton X-100 (24 ​h) used as the positive control. Data are expressed as mean ​± ​standard deviation (n tests ​= ​3) and showed as percentage relative to the preexposure TEER values of each tissue. ∗p ​< ​0.05 vs. untreated, control cells (CTRL). Confocal Laser Scanning Microscopy (CLSM) images of (S) control human bronchial epithelial cells after 30 days of culture and (T) cells exposed repeatedly for 30 days to aerosolized GO (scale bars: 10 ​μm). Cells were stained with ZO-1 antibody (tight junction protein, in red). TEM, transmission electron microscopy; GO, graphene oxide; 3D, three-dimensional; SEM, scanning electron microscopy; LDH, lactate dehydrogenase; n, nucleus; v, vesicle.

Viability of cells was also monitored using two biochemical assays: the resazurin assay for assessing the cellular mitochondrial activity [23] and the LDH assay for membrane damage [45]. As shown in Fig. 4A–C, both the assays indicated that prolonged exposure to GO did not induce any cytotoxic effects to airway tissue even after 30 days of exposure, as opposite to the positive control that induced a strong viability decrease after 24h of exposure (Fig. 4B–D). In addition, no morphological alteration of the cellular surface (on which ciliated and globet cells are exposed) was observed, as reported by SEM (Fig. 4–H) and confocal microscopy analyses (Fig. 4I–L).

Overall, these results indicated that prolonged exposure to GO for 30 days did not affect the viability and integrity of the airway tissue. To our knowledge, only one study analyzed the exposure to low aerosolized doses of GO using a 3D airway model, although under acute exposure, using a single aerosolization process [14]. In line with our data, this article reported no changes in cell viability and morphology after a single exposure to GO aerosol in a dose range of 0.84–1.02 ​μg/cm^2^, which approximately corresponds to the dose applied to our first day of exposure (0.715± ​0.05 ​μg/cm^2^).

To compare the 3D airway model response to a 2D cell system, the same exposure setup (i.e. ​the ALI condition and the prolonged GO exposition, refer experimental) was applied to BEAS-2B bronchial cell line (Fig. 4M–P). As shown, repeated exposure to GO produced a significant decrease in cell viability in a time- and dose-dependent manner. This result was confirmed by TEM images (Fig. 4O–P) showing remarkable alteration of cell morphology after 15 days of exposure to GO aerosol. These data highlight the importance of using physiologically relevant *in vitro* models to predict the impact of real-world human exposure to inhaled particles ​because the use of airway cell lines (2D systems) may overestimate toxicity of nanoparticles due to the absence of mucociliary clearance [46,47]. The lower efficiency of this process may cause an increase of the GO uptake process, with an increase of its effective intracellular dose. Therefore, 3D systems are likely to offer an increasingly attractive substitute compared with 2D cell culture.

### 3.4 Repeated aerosol exposure to GO induces impairment of airway barrier integrity

The physical barrier of the lung includes, together with the mucociliary apparatus, the intercellular junctions. Defined as TJs, they form adhesive forces that connect neighboring cells, separating the external environment from the subepithelial tissue [48]. The TJ damage is the major cause of epithelial barrier breakdown during lung inflammation [49]. In the present work, we assessed the barrier integrity of the 3D airway model exposed to 30 repeated GO aerosolization by measuring the TEER of the barrier (Fig. 4Q–R). Because it is considered a very sensitive and reliable method to confirm the integrity and permeability of the lung barrier model [50], TEER was monitored for all the used exposure times (0–30 days), and results were expressed as percentage relative to the preexposure TEER values of each tissue (Fig. 4R). Table S3 shows the TEER values not normalized with the preexposure value ​to demonstrate that the tissues maintained good barrier function during overall the experiment. Indeed, control cell ​values are well above the 300 ​Ω ​cm^2^ cut-off reported by manufacturer's instructions. Results indicate that, after about 20 days of exposure to GO, a moderate decrease of TEER is detected. In particular, a 40% TEER decrease was found at the time of 25-repeated exposure times, reaching a plateau up to the end of the treatment (Fig. 4Q). TEER reduction can be caused by uncontrolled cell death within the layer [51] or also by other subtle phenomena, such as the alteration of transcellular ion flux caused by signaling or physiology damage of the paracellular barrier (e.g. claudin expression patterns or other proteins) [52]. Based on our data, we can exclude a physiological damage to the epithelial barrier, as we did not report ​any modifications in TJs after 30-repeated exposure times to GO compared with the negative control cells (ZO-1 expression, Fig. 4S–T). In addition, TEER decrease could not be ascribed to cell death as the viability and integrity of the 3D airway were maintained unaltered till the end of the treatment (Fig. 3, Fig. 4A–L). We thus hypothesize that some specific perturbations eliciting changes in transcellular ion flux occur. TEER alteration in absence of viability changes was reported in many cell systems [[21], [53], [55]]. Notably, Bramini et al.[21] reported such an effect for GO exposure, demonstrating that chronic treatment of primary cortical neurons with GO altered Ca^2+^ dynamics and homeostasis without major alterations of cell viability. This effect is only visible during chronic treatments and not evidenced by acute studies. Similarly, our data suggest subtle alterations of plasma membrane channels or pumps, due to the repeated treatment of the 3D airway model with GO. Because a potential dysfunction of TJ proteins could contribute to the pathogenesis of a variety of inflammatory lung diseases [30], further studies are required to substantiate this hypothesis.

### 3.5 GO aerosol exposure induces a proinflammatory response without triggering oxidative stress

**Fig. 5:**
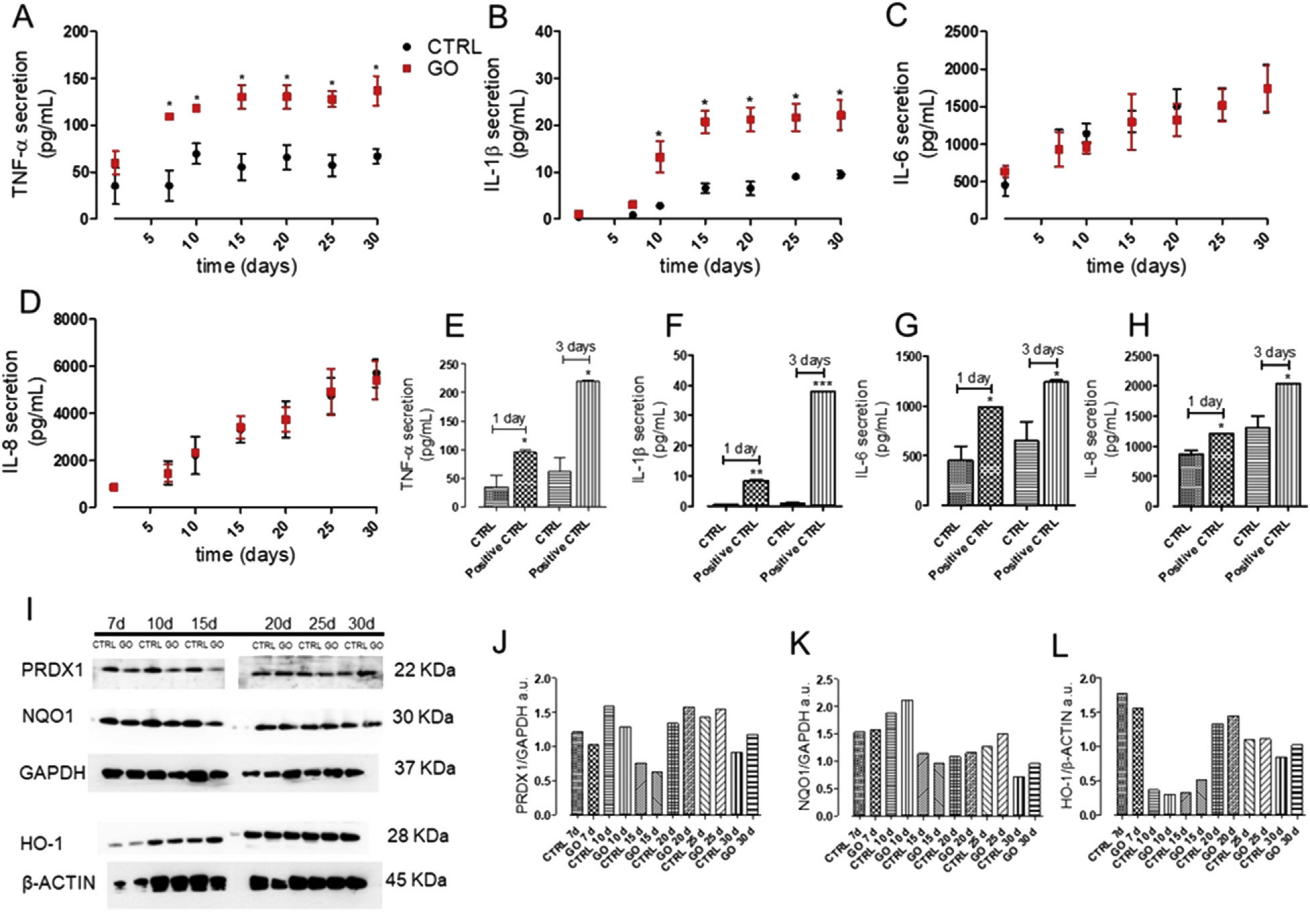
Inflammatory and oxidative stress response of 3D airway model exposed to GO aerosol. Release of accumulated (A) TNF-α, (B) IL-1β, (C) IL-6, and (D) IL-8 into basolateral compartment of tissues. (E-F-G-H) Release of the same cytokines by cells treated for 3 days, basolaterally with 10 ​ng/mL TNF-α, used as the positive control. Data are expressed as mean ​± ​standard deviation (n tests ​= ​3). ∗p ​< ​0.05, ∗∗p ​< ​0.01 and ∗∗∗p ​< ​0.001 vs. untreated, control cells (CTRL). (I-J-K-L) Western blot of proteins involved into the antioxidant stress response (PDRX1, NQO1, and HO-1). Representative blots are shown. Quantification of western blotting was performed with Image J software (NIH, USA). GAPDH expression was reported as protein loading control. The experiment was performed twice with comparable results. GO, graphene oxide; 3D, three-dimensional; TNF-α, tumor necrosis factor alpha; IL-1β, interleukin-1β; IL-6, interleukin-6; IL-8, interleukin-8; NQO1, NAD(P)H quinone dehydrogenase 1; HO-1, heme oxygenase-1.

*In vivo* inhalation toxicity studies (that used aerosol as exposure mode) reported that GFMs (including GO) induce minimal and reversible pulmonary toxicity and inflammation [[15], [56], [57]]. However, none of these works were performed under chronic or subchronic experimental conditions. On the other hand, most of the in vitro data (using 2D immortalized confluent cells) show that GO promotes ROS induction, resulting in oxidative stress and inflammation [[15], [56], [57]]. As opposite to these findings, however, in a 3D human airway model, neither oxidative stress nor secretion of proinflammatory markers (IL-8, IL-1β, and TNF-α) after 1 day of acute exposure were reported [14]. In our study, we aimed at monitoring the inflammatory response of the 3D airway model upon repeated treatments with GO, at doses relevant for worker occupational exposure. We quantified the secretion of TNF-α, IL-6, IL-8, and IL-1β ​that act as key mediators of inflammation/immune response of airway epithelial cells, upon an external insult [[48], [58]]. As reported in Fig. 5A and B, results indicated that GO-repeated exposure induced a significant, time-dependent increase of TNF-α and IL-1β secretion compared with the control. However, no increased secretion of IL-6 and IL-8 was registered (Fig. 5C and D). Moreover, we observed a lack of oxidative stress response. PRDX1, NQO1, and HO-1, which are cytoprotective enzymes activated by the nuclear factor (erythroid-derived 2)–like 2 pathway in the oxidative cellular damage [59], were indeed not found altered compared with the control cell level (Fig. 5I–L). Overall, our data, showing slightly increased secretion of TNF-α and IL-1β, not accompanied by oxidative stress ​suggest that the GO-induced inflammation upon 30 repeated exposure is too weak for the activation of the inflammatory cascade. At the first dose used (1 day, 0.7 ​μg/cm^2^), our results are in line with Drasler et al. [14], which are related to an acute response. However, it is worth noting that incremental doses of GO (as developed in our experimental design for 1 month) correspond to the GO exposition levels of real worker lifetime. This thus reveals that some proinflammatory response can be likely in a worker exposed to GO for his entire lifetime.

On the other side, IL-1β follows a different pathway that is critically regulated by cytosolic molecular complexes, termed inflammasomes, such as NLRP3 [60,61]. The activation of NLRP3 has been reported in various mammalian cell types, such as airway epithelial cells, in response to diverse stimuli, including microbes, viral RNA, ATP, uric acid crystals, environmental particles, and fibers [62]. Notably, excessive or prolonged IL-1β secretion is associated with numerous acute and chronic inflammatory diseases, including asthma and chronic obstructive pulmonary disease [[61], [62], [63], [64]]. Although our data did not focus on NLRP3 expression, in our system it is reasonable to relate the induction of IL-1β to the inflammasome activation. This hypothesis is supported by literature data that report that, GO triggers IL-1β production in macrophages and lung epithelial cell lines and that the cellular GO uptake is the initial event for IL-1β production. We indeed found a time correlation between the increase of IL-1β and the GO uptake, both occurring at about 15 days of GO-repeated exposure (Fig. 3C and D and Fig. 5B). Furthermore, it is known that TNF-α and IL-1β are strictly connected because TNF-α can enhance IL-1β secretion and vice versa [65]. Notably, these two cytokines together play an important role in the development of airway hyper sensibility, for example, asthma [[65], [66], [67]], so that inhalation of GO should be considered with attention, as individuals with asthma may be more susceptible to its adverse effects.

### 3.6 Repeated aerosol GO exposure produces a transient blockade of bronchial autophagy

**Fig. 6:**
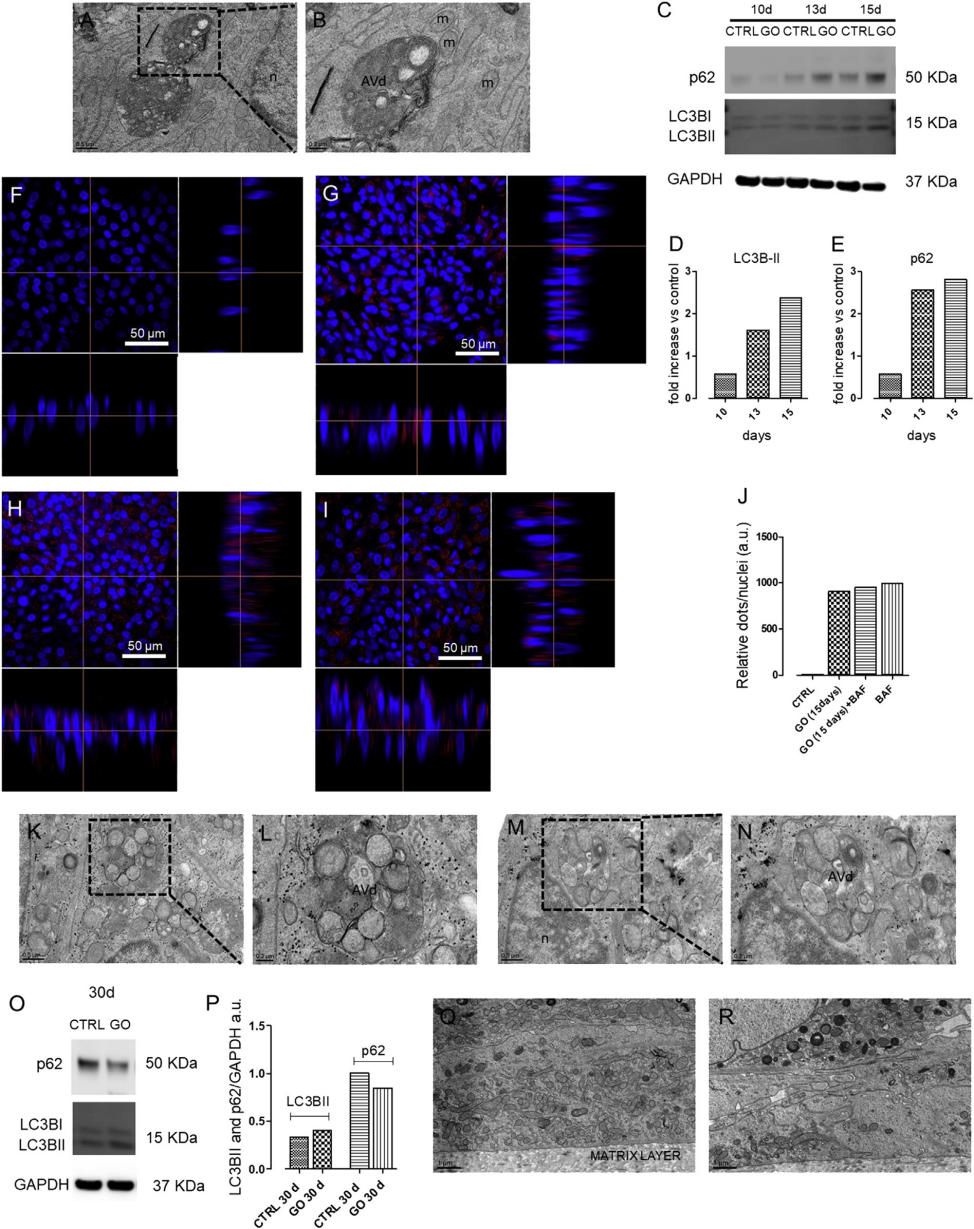
Autophagy induction by bronchial cells exposed repeatedly to GO aerosol. (A–B) Representative TEM micrographs of (A–B) cells exposed to GO for 15 days (scale bars:0.5 ​μm ​at lower magnification and 0.2 ​μm ​at higher magnification). (C-D-E) Immunoblot analysis and quantification of LC3B and p62 in cells exposed to GO aerosol up to 15 days. (F-G-H-I) Representative z-sectioning confocal microscopy images of (F) control cells, (G) cells exposed to GO for 15 days, (H) cells exposed to GO in combination with BAF (GO+BAF), and (I) cells treated with BAF alone. Cells were stained with Hoechst 33342 (nuclei, in blue) and LC3B antibody (autophagic marker, in red). Scale bars: 50 ​μm (63 ​× ​objective lens). (J) Relative quantification of LC3 dots, performed with Image J software. (K-L-M-N) Representative TEM micrographs of (K–L) cells exposed to GO in combination with BAF and (M–N) cell exposed with BAF alone (scale bars:0.5 ​μm ​at lower magnification and 0.2 ​μm ​at higher magnification). (O–P) Immunoblot analysis of LC3B and p62 in cells exposed to GO aerosol up to 30 days. For western blot analysis, representative blots are shown. Quantification of western blotting was performed with Image J software. GAPDH expression was reported as protein loading control. The experiment was performed twice with comparable results. (Q–R) Representative TEM micrographs of (O) control tissue and (P) GO-treated cells up to 30 days (scale bar: 1 ​μm). TEM, transmission electron microscopy; GO, graphene oxide; BAF, bafilomycin; m, mitochondria; n, nucleus; AVd, late/degradative autophagic vacuoles.

Macroautophagy (hereafter referred to as autophagy) is a cellular recycling pathway that plays a crucial role in adaptive responses to nutrient deprivation and other forms of stress [[56], [68], [70]]. Autophagy process involves a proteosomal-independent degradative mechanism in which cytoplasmic material is engulfed in double membrane vesicles, named autophagosomes, and delivered to lysosomes for degradation [71]. Although several studies in cultured cells reported a GO-mediated induction of autophagy, as summarized by Ou el al. [71], autophagy has been never studied in a complex 3D model in relation to hazard assessment of graphene materials. Notably, a new TEM analysis of the 3D airway tissue exposed to aerosolized GO for 15 days showed a cytoplasmic accumulation of vesicular structures (Fig. 6A and B), already shown in Fig. 3. Magnified TEM image of these vesicles revealed typical morphological features of late/degradative autophagic vacuoles (AVd) (Fig. 6B). Indeed, the ultrastructure of the vesicular internal content showed a partially or completely degraded cytoplasmic material that appears strongly electron dense (dark) in OsO_4_ ​post-fixed TEM sections. Remarkably, accumulation of AVd is generally observed upon a blockade of the autophagy process at the late stage (i.e. ​autophagosome maturation), suggesting that prolonged GO exposure may impair the autophagic flux of bronchial cells. To evaluate this possibility, we performed combined biochemical and confocal microscopy analyses in the 3D airway model. During autophagosome biogenesis, a cytosolic form of LC3 (LC3-I) is converted to a lipidated LC3-II that is specifically recruited on the membrane of autophagosomes [72]. Hence, variations in autophagosome turnover upon diverse treatments can be monitored by evaluating cellular LC3-II levels with immunoblot assays [72]. We thus assessed LC3-II protein levels after 10, 13, and 15 days of GO treatment using a specific anti-LC3B antibody that has a higher affinity for this form [[68], [69], [70]]. Results clearly indicated that GO-treated cells showed a time-dependent increase in LC3-II levels compared with the control, indicating a GO-mediated modulation of cellular autophagy (Fig. 6C and D). We next evaluated the levels of the autophagic receptor SQSTM1, also known as p62. SQSTM1 and SQSTM1-bound polyubiquitinated proteins are degraded in autolysosomes, thus serving as an index of autophagic degradative flux [68]. Consistent with a GO-mediated inhibition of autophagy, 3D airway tissue showed an increasing accumulation of p62 levels in samples treated for 10, 13, and 15 days with GO, compared with the control (Fig. 6C–E).

**Fig. 7:**
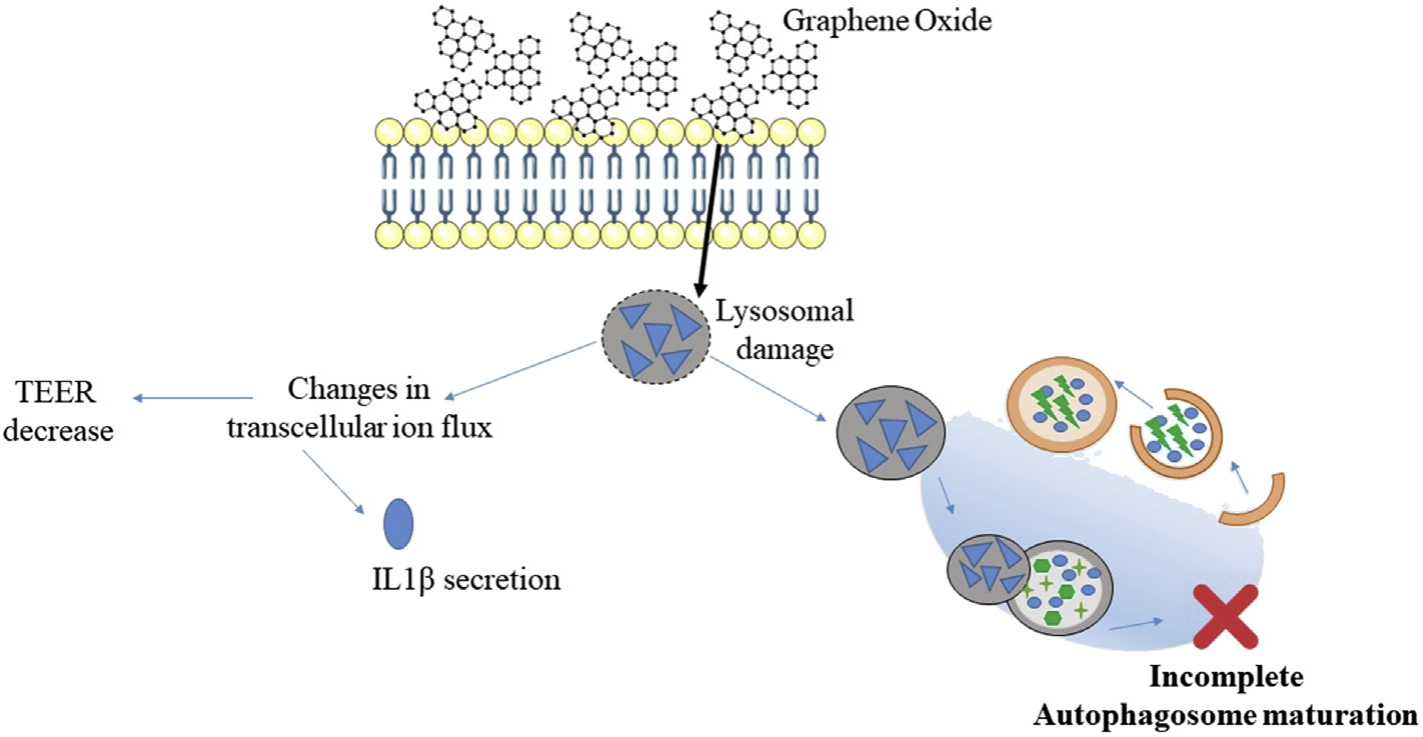
Schematic representation of the molecular mechanism behind the autophagy blockage.

To confirm the GO-mediated impairment of autophagy, we conducted confocal immunofluorescence microscopy in our bronchial model exposed to GO for 15 days. As lipidated LC3 is recruited on the autophagosomal membrane from the initial stages of autophagy, LC3-II–containing autophagosomes appear as perinuclear fluorescent dots when assessed by indirect immunofluorescence. Consistent with our immunoblot analysis, cells exposed to GO showed a higher number of fluorescent autophagosomes than the control (Fig. 6F and G). To further support an autophagic inhibitory activity of GO, 3D airway tissue treated with aerosol GO exposure or water (negative control) for 15 days received a further day of treatment in presence of the late-stage autophagy inhibitor, BAF A1BAF ​[69]. Fully consistent with a GO-mediated inhibition of bronchial autophagic flux, GO-treated, GO+BAF-treated, and BAF-treated samples showed negligible differences in the number of fluorescent perinuclear dots (Fig. 6G–J). In addition, also at TEM level, we did not reported differences in term of structure of the AVd vacuoles between the same cell groups (Fig. 6A and B and Fig. 6K–N). Collectively, our data indicate that prolonged aerosol GO exposure inhibits autophagy by preventing autophagolysosome maturation. A lysosomal-dependent blockade of autophagy may arise from an impairment of lysosome acidification by (1) inhibition of lysosomal ATPases (e.g. BAF A1) [69], (2) lysosomal pH reduction by lysosomotropic agents (e.g. chloroquine) [73], or (3) lysosomal membrane permeabilization (e.g. L-leucyl-l-leucine methyl ester) [74]. Considering the size and the weak acidic/neutral properties of GO [75], it is likely that an impairment of lysosomal membrane is behind the GO-mediated inhibition of autophagolysosome function. Notably, lysosomal membrane permeabilization has been reported to induce an inflammasome-mediated activation of IL-1β and changes in ion flux [76], which is consistent with the observed induction of this cytokine in 3D bronchial model upon GO treatment (Fig. 5B) and with TEER decrease (Fig 4Q). The scheme in Fig. 7 summarizes this mechanism. Moreover, immunoblot analysis from organotypic cultures exposed to aerosol GO longer than 15 days showed minor differences in the levels of LC3-II and p62 proteins between treated and control samples (Fig. 6O–P). In line with this concept, TEM analysis from bronchial cells treated with GO for 30 days did not reveal a significant accumulation of autophagolysosomes (Fig. 3G and H and Fig. 6Q–R), suggesting that cells can overcome defects in autophagy over a long period. ​We also showed the absence of perinuclear fluorescent dots (LC3-II-containing autophagosomes) by immunofluorescence in 30 day-treated GO tissue (Fig. S4). The recovery of a functional autophagosomal activity correspondeds to a marked decrease in GO uptake (Fig. 3G and H), suggesting that cells can overcome defects in autophagy over a long period by reducing GO intracellular accumulation and allowing the synthesis of novel functional autophagolysosomes. Albeit this adaptive response can restore a physiological autophagic flux, our data indicate that repeated GO exposure will generate a time window within which bronchial autophagy is strongly impaired. Autophagy represents a fundamental adaptive response to diverse stressors, including pathogens and environmentally induced oxidative stress [77], which represent noxious stimuli to which lungs are continuously exposed. Consequently, GO-mediated autophagy inhibition may potentially increase susceptibility to pulmonary infections and lung diseases. In this context, the model introduced here provides a relevant tool for assessing the hazard potential of GO-related autophagic dysfunction under experimentally induced pathological conditions (e.g. pulmonary pathogenic bacteria, particulate pollutants, or nicotine).

## 4 Conclusion

By means of a nebulizer system, using doses (0.7–20 ​μg/cm^2^), which reflect a real worker exposure lifetime to GO in a production facility, a 3D airway model was repeatedly exposed to GO for 4 weeks, and the effects exerted were followed at short term (every 24 ​h). Overall, our results highlighted the importance of using physiologically relevant in vitro airway models to predict the impact of real-world exposure to inhaled nanoparticles compared with 2D systems, which lack of mucociliary clearance and, consequently, could have higher particle uptake. Notably, experimental data indicated that, at the conditions used in this study, 30-day repeated exposure to GO did not elicit strong toxic effects at short term, as opposite to a 2D cell system, based on BEAS-2B bronchial cell line. However, despite the efficient clearance operated by the mucociliary apparatus and the lower uptake typical of the lung non-phagocytic cells, few internalized GO structures appeared translocated in the basolateral layer at the end of the treatment (after 30 days). This finding suggests that GO is not completely eliminated by the mucociliary clearance system of 3D bronchial cells, thus highlighting the importance to select properly the cell model for its toxicity evaluation. In a realistic human exposure scenario, where prolonged exposure of workers to GO in occupational settings may occur, the GO degradation/accumulation must be indeed evaluated carefully. We can hypothesize that the GO accumulation during the occupational worker lifetime will allow for the material translocation through the lower region of the lung, that is, the alveoli, where the clearance by alveolar macrophages is expected after months or years. Further studies are needed to understand the long-term impact of GO on the alveolar epithelial barrier.

In addition, after two weeks of GO exposure (ca. 10 ​μg/cm^2^), a persistent proinflammatory response (stimulation of TNF-α and IL1β, not accompanied by oxidative stress) coupled to barrier impairment was detected. Lastly, at prolonged aerosol GO exposure, we also observed a late-stage autophagy blockage. Interestingly, cells seem to overcome defects in autophagy over a long period, as suggested by a reduction in the number of AVd and a correspondent decrease of GO uptake at 30 days. The presence of a time window in which the autophagy mechanism is impaired, together with prolonged proinflammatory effects, may suggest that inhalation of GO should be considered with high attention, as workers with pulmonary infections and/or lung diseases may be susceptible to its adverse effects.

In the future, the perspective of an in vitro/in vivo validation of the presented 3D airway model will pave the way toward its use in precautionary occupational contexts. The model, when validated, may indeed provide risk assessment–oriented data (i.e. NOEL) to facilitate risk classification of emergent nanomaterials.

## Author contributions

L.D.C., S.S., B.G., and P.P.P., conceived the study. L.D.C. performed the experiments. T.C. imaged and processed transmission electron microscopy samples. E.V. provided characterization studies on graphene oxide. L.D.C. and S.S. wrote the article. All revised the article.

## Conflict of interest statement

The authors declare that they have no known competing financial interests or personal relationships that could have appeared to influence the work reported in this paper.
