# Supplementary material for: Repeated exposure to aerosolized graphene oxide mediates autophagy inhibition and inflammation in a three-dimensional human airway model
Source: Mater Today Bio. 2020 Mar 24;6:100050. doi: 10.1016/j.mtbio.2020.100050 (PMC7171197; doi:10.1016/j.mtbio.2020.100050)
Supplement: Multimedia component 1 [file mmc1.docx]

REPEATED EXPOSURE TO GRAPHENE OXIDE BY AEROSOL MEDIATES AUTOPHAGY INHIBITION ALONG WITH INFLAMMATION IN A 3D HUMAN AIRWAY MODEL

Luisana Di Cristo, ^1^ Benedetto Grimaldi,^1^ Tiziano Catelani,^2^ Ester Vázquez ^3^, Pier Paolo Pompa,^4^and Stefania Sabella^1^

^1^ Drug Discovery and Development Department, Istituto Italiano di Tecnologia, Via Morego, 30 – 16136 Genova, Italy

^2^ Electron Microscopy Facility, Istituto Italiano di Tecnologia, Via Morego 30 – 16163 Genova, Italy

^3^ Departamento de Química Orgánica, Facultad de Ciencias y Tecnologías Químicas-IRICA, Universidad de Castilla-La Mancha, 13071 Ciudad Real, Spain,

^4^ Nanobiointeractions&Nanodiagnostics, Istituto Italiano di Tecnologia (IIT), Via Morego, 30 – 16163 Genova, Italy

**Supporting Information**


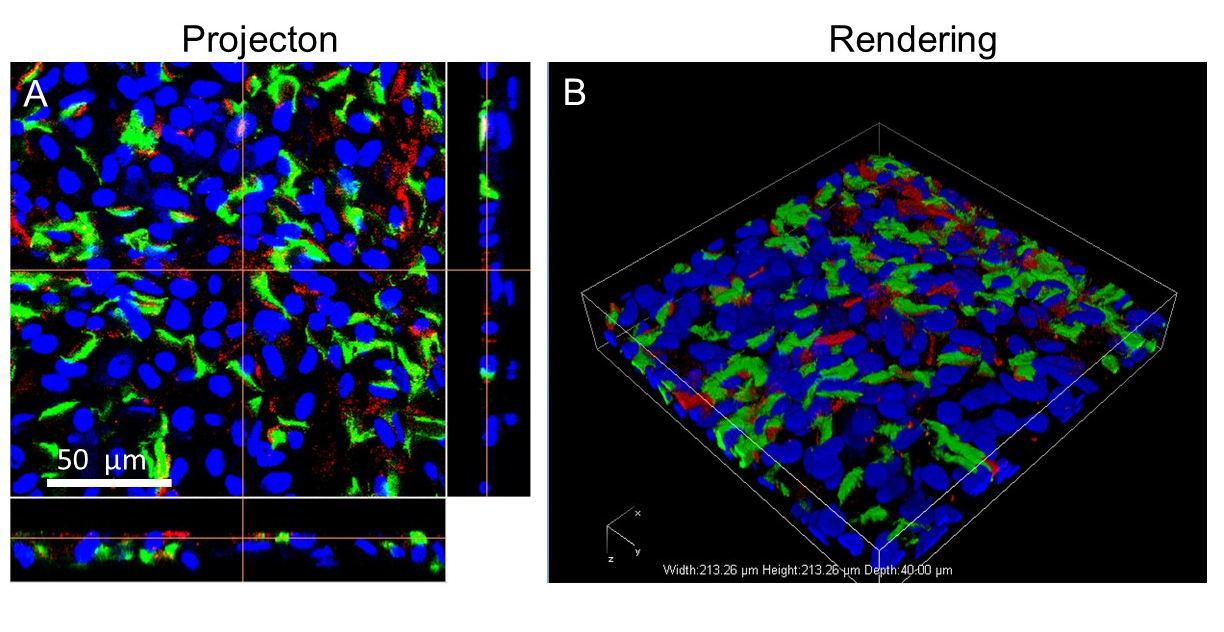


**Figure S1**. LSCM images of 3D airway model at time zero (T0) (one day before starting the experiment). Cells were stained with Hoechst 33342 (nuclei, in blue), Mucin 5AC antibody (globet cells, in red) and alpha Tubulin (acetyl K40) antibody (cilia cells, in green). Scale bars: 50 μm (63× objective lens).

| **Ag (μg/l)** | <1 |
| --- | --- |
| **Al (μg/l)** | <50 |
| **Ba (μg/l)** | 24 |
| **Cd (μg/l)** | <1 |
| **Co (μg/l)** | 2 |
| **Cr (μg/l)** | 4 |
| **Cu (μg/l)** | 256 |
| **Fe (μg/l)** | <50 |
| **Hg (μg/l)** | <5 |
| **La (μg/l)** | <0,1 |
| **Mn (μg/l)** | 2182 |
| **Mo (μg/l)** | 8 |
| **Ni (μg/l)** | <10 |
| **Pb (μg/l)** | 18 |
| **Pd (μg/l)** | <0,1 |
| **Ru (μg/l)** | <0,5 |
| **Se (μg/l)** | <0,1 |
| **Ti (μg/l)** | <10 |
| **W (μg/l)** | 7 |
| **Zn (μg/l)** | 334 |

**Table S.2.** Metallic-impurities content of GO suspension measured by Inductively Coupled Plasma Mass Spectrometry (ICP-MS) technique.

**Table S3**. Average of TEER values of CTRL and GO- treated tissues obtained at each tested time points. Data are expressed as mean ± standard deviation (n tests = 3).


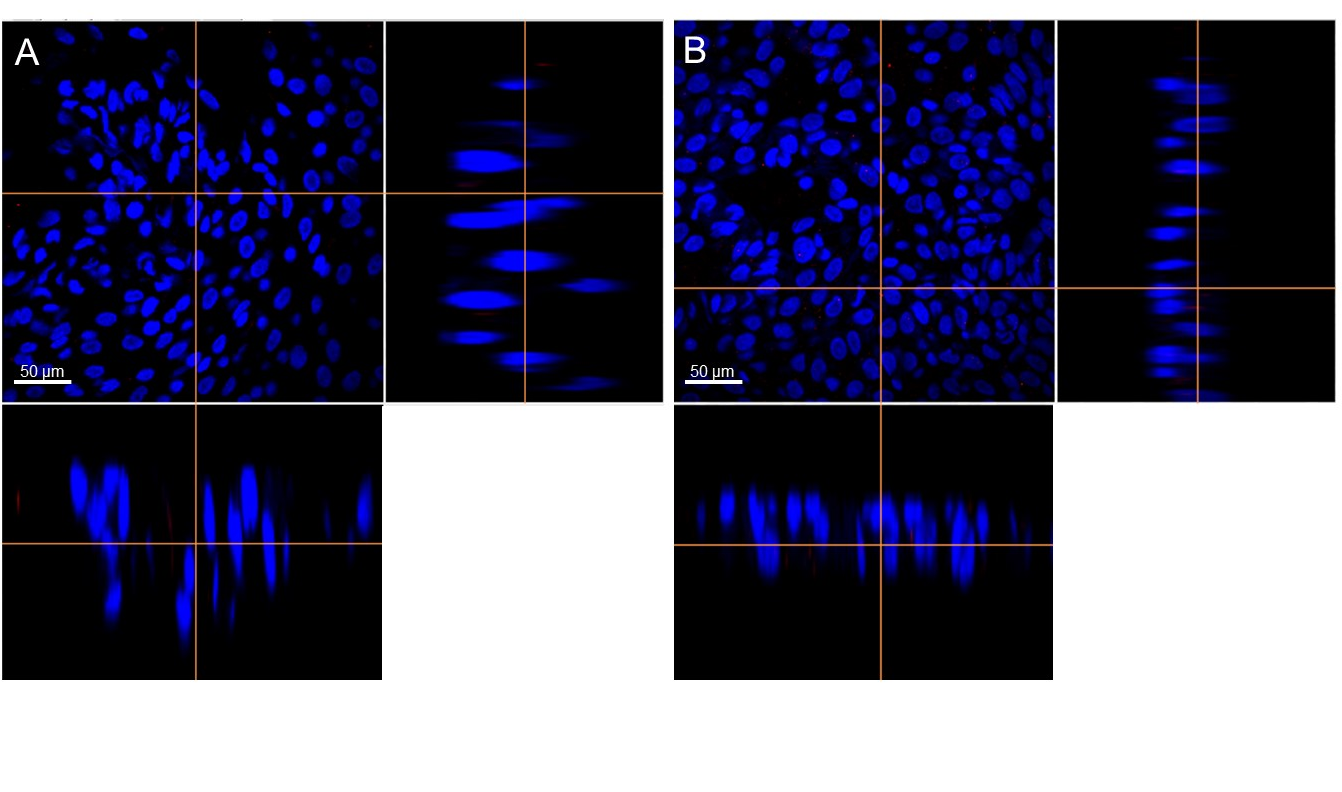


**Figure S4.** LSCM images of 3D airway model exposed to GO for 30 days. (A) Representative z-sectioning confocal microscopy images of control cells after 30 days of cultures and (B) cells exposed to GO at 30 days. Cells were stained with Hoechst 33342 (nuclei, in blue) and LC3B antibody (autophagic marker, in red). Scale bars: 50 μm (63× objective lens).
